# Clinical characteristics of bacterial vaginosis among women testing positive for fastidious bacteria

**DOI:** 10.1136/sti.2008.032821

**Published:** 2008-11-12

**Authors:** C L Haggerty, P A Totten, M Ferris, D H Martin, S Hoferka, S G Astete, R Ondondo, J Norori, R B Ness

**Affiliations:** 1 University of Pittsburgh, Pittsburgh, Philadelphia, USA; 2 Department of Medicine, Division of Infectious Diseases, University of Washington, Seattle, Washington, USA; 3 Research Institute for Children, Departments of Pediatrics and Microbiology, LSUHSC, New Orleans, Louisiana, USA

## Abstract

**Objectives:**

As the aetiology of bacterial vaginosis (BV) is not well understood, this study sought to determine the relationships between several fastidious microbes, BV and selected clinical characteristics of BV.

**Methods:**

Endometrial and cervical specimens from 50 women with non-gonococcal, non-chlamydial endometritis were tested for *Leptotrichia sanguinegens/amnionii*, *Atopobium vaginae*, bacterial vaginosis-associated bacteria 1 (BVAB1), *Ureaplasma* *urealyticum* biovar 2 (UU-2) and *Ureaplasma parvum* using PCR. BV was categorised using Nugent’s and Amsel’s criteria. Odds ratios (OR) adjusted for age and race were estimated using multivariable logistic regression.

**Results:**

Although elevated pH was a universal feature, other BV characteristics differed by pathogen, suggesting variable clinical presentation. Only UU-2 was strongly associated with vaginal discharge, but a positive whiff test and a 20% or greater classification of epithelial cells as clue cells were more common among women with *L sanguinegens/amnionii*, *A vaginae* and BVAB1. For each of these bacteria, there were trends towards associations with BV defined by Amsel’s criteria (*L sanguinegens/amnionii* OR 2.9, 95% CI 0.5 to 15.7; *A vaginae* OR 2.6, 95% CI 0.6 to 11.4; BVAB1 OR 5.7, 95% CI 1.0 to 31.1) and significant associations with BV defined by Gram stain (*L sanguinegens/amnionii* OR 17.7, 95% CI 2.8 to 113.0; *A vaginae* OR 19.2, 95% CI 3.7 to 98.7; BVAB1 OR 21.1, 95% CI 2.2 to 198.5).

**Conclusions:**

*L sanguinegens/amnionii*, *A vaginae* and BVAB1 are associated with clinical characteristics consistent with BV and BV defined by Nugent’s and Amsel’s criteria. These fastidious bacteria may cause unrecognised infection, as none was associated with abnormal vaginal discharge.

Bacterial vaginosis (BV) is a common lower genital tract infection that may lead to pelvic inflammatory disease (PID),^1^ subsequent infertility^2^ and preterm birth,^3^ and may increase susceptibility to HIV.^4^ Although no single agent is known to cause BV and its aetiology is not well understood, bacteria such as *Gardnerella vaginalis*, ureaplasmas, *Mycoplasma hominis* and anaerobic bacteria are commonly isolated from BV patients.^5^^–^7 Until recently, our understanding of BV microbiology largely came from cultivated species and Gram-stained vaginal smear analysis, most notably Nugent’s criteria.^8^ Recently, cultivation-independent studies using 16S rDNA sequences PCR amplified from vaginal DNA have revealed previously unrecognised bacterial genera associated with BV. Some of these include metronidazole-resistant^5^ *Atopobium vaginae*,^5^ ^6^ ^9^ *Leptotrichia (Sneathia) sanguinegens* and *Leptotrichia* *amnionii*,^6^ and three new species of uncultivated bacteria termed bacterial vaginosis-associated bacteria (BVAB) types 1, 2 and 3.^6^

Nugent’s criteria^8^ measure specific Gram-stained bacterial morphotypes. Consequently, organisms that do not Gram stain, such as ureaplasmas and mycoplasmas, are not measured. Ureaplasmas have been associated with BV,^10^ although modestly in some studies.^7^ High rates of ureaplasma colonisation among patients without clinical disease further complicate interpretation.^10^ ^11^ Moreover, ureaplasmas (formerly designated *Ureaplasma urealyticum* but termed undifferentiated ureaplasmas herein) have recently been split into two biovars: *Ureaplasma parvum*, reported to be non-pathogenic in men;^12^ and *U urealyticum* (biovar 2), associated with urethritis.^12^ ^13^ The role of these newly classified ureaplasmas in BV has been little studied. In this analysis, we sought to determine the associations among several fastidious pathogens and BV defined by Gram stain and Amsel’s criteria among a population of women with PID.

## MATERIALS AND METHODS

### Population

A subset of previously collected and stored cervical and endometrial specimens from women who participated in the PID Evaluation and Clinical Health (PEACH) Study^14^ was analysed. Briefly, the parent PEACH study recruited 831 women aged 14–37 years with clinically suspected PID between March 1996 and February 1999 from emergency departments, obstetrics and gynaecology clinics, sexually transmitted infection (STI) clinics and private practices at 13 US clinical sites. All women gave written informed consent, and University of Pittsburgh Institutional Review Board approval was obtained for both the PEACH study and the substudy described herein. In order to examine the lower and upper genital tract microbiological milieu among women with non-gonococcal, non-chlamydial PID, and additionally to examine the associations between selected fastidious bacteria and BV, we conducted a targeted PCR substudy using stored specimens from 50 randomly selected women with histologically confirmed non-gonococcal, non-chlamydial endometritis. A classification of endometritis was given upon finding at least five neutrophils in the endometrial surface epithelium in the absence of menstrual endometrium and/or at least two plasma cells in the endometrial stroma.^15^

### Microbiological studies

As part of the current substudy, previously frozen endometrial biopsy and cervical swab specimens were tested for *L sanguinegens/amnionii*, *A vaginae*, BVAB1, *U urealyticum* (biovar 2), and *U parvum* using PCR assays. Patient specimens were purified using the MasterPure DNA purification kit (Epicentre, Madison, Wisconsin, USA). For the *Leptotrichia* PCR assay, the published *Leptotrichia*-specific primers^6^ were optimised for our thermocycling conditions. *Leptotrichia*-specific PCR products were detected as approximately 240 bp products on agarose gels. The specificity of this assay was confirmed by sequencing the PCR products from four randomly selected specimens; two of the specimens contained sequences consistent with *L amnionii* and two contained sequences consistent with *L sanguinegens*.

PCR primers targeting the 16S rRNA genes of *A vaginae*^5^ and BVAB1^6^ were 5′–GTTAGGTCAGGAGTTAAATCTG–3′ and 5′–TCATGGCCCAGACC–3′ and 5′–AGTGTAGGCGGCACTATAAG–3′and 5′–CGATAACTGACGCTAAGGCT–3′, respectively. The PCR conditions were 95°C (5 minutes) followed by 25 cycles of 95°C (1 minute), 62°C (1 minute) *A vaginae* or 66°C (1 minute) for BVAB1, 72°C (1 minute) with final extension 72°C for 7 minutes. PCR products (150–200 bp) were visualised on agarose gels sequenced to confirm specificity.

The *U urealyticum* (biovar 2) PCR assay was designed to differentiate the newly recognised species of ureaplasma, *U urealyticum* and *U parvum*, formally called *U urealyticum* biovar 2 and biovar 1, respectively. The *U urealyticum* PCR targets sequences in the urease genes of all 10 serovars of UU-2 not found in the four *U parvum* (biovar 1) serovars. Conversely, the *U parvum* PCR targets sequences in the urease genes of all *U parvum* serotypes not found in the UU-2 serovars. In both assays, 5 μl purified patient sample was subjected to PCR amplification in a total volume of 100 μl containing 1 × PCR buffer (magnesium ion free; Promega, Madison, Wisconsin, USA), 2.75 mmol magnesium chloride, 200 μmol each of the four dNTP, 0.1 μmol forward primer, 0.1 μmol reverse primer and 5 U of Taq DNA polymerase. PCR primers and amplification conditions for the *U urealyticum* PCR were: UUF2 (5′–CAC AGA TGT CCT TGA TGT ACC C–3′) and UUR2 (5′–GTA AAA ATT ATT TGT AAA TTG GGC–3′); and the following temperatures: 94°C for 4 minutes; 45 cycles of 94°C for 10 s, 56°C for 50 s and 72°C for 30 s; followed by 72°C for 5 minutes and a final soak set at 4°C indefinitely. Primers and conditions for the *U parvum* PCR were UpF1 (5′–GATCCAATTTACAAATAACA–3′) and UpR1 (5′–GTAGAAACTTGTAAAATAGAG–3′); and 94°C for 4 minutes; cycle programme (35 cycles); 94°C, 45°C and 72°C for 1 minute each; followed by 72°C for 10 minutes and a final soak set at 4°C indefinitely. Assay specificities were confirmed by the detection of the appropriate sized PCR product (474 and 469 for *Uurealyticum* and *U parvum*) on agarose gels and reactivity with the UU-2-specific probe (UUDP2 (5′–biotin–ATC CAA AAG TTA TTG GTA ACA GTA G–3′) and *U parvum* (5′–biotin–GGCAATTAATTTCGCTAGT–3′)-specific probes on Southern blots hybridised at 55°C and 50°C, respectively, and visualised on Kodak BioMax XAR film after reaction in the chemiluminescence assay using the ECL kit (Amersham GE Healthcare, Little Chalfont, UK).

In a subset of 352 women in the PEACH study, endometrial biopsy tissue specimens were examined for anaerobic Gram-negative rods, *G vaginalis* and *M hominis*. Twenty-nine women in the current substudy had culture results. One sample was used to inoculate a human blood bilayer Tween agar^16^ for the detection of *G vaginalis*, and a second swab sample was used to inoculate a Brucella agar for recovery of anaerobic bacteria and broth media for *M hominis* recovery.

Vaginal fluid was analysed for bacterial vaginosis by Amsel’s criteria^17^ and by Nugent’s criteria, in which the relative presence of morphotypes consistent with *Lactobacillus* spp, *Gardnerella* spp, *Mobiluncus* and anaerobic bacteria is measured by Gram stain with a score ranging from 0 to 10.^8^

### Statistical analysis

Comparisons of demographic, sexual and behavioural characteristics, and cultivable comorbid BV bacteria were compared between women with and without *L sanguinegens/amnionii*, *A vaginae*, BVAB1, UU-2 and *U parvum* using χ^2^ tests of proportions and Fisher’s exact tests. Logistic regression models were fit with BV as the dependent variable and *Leptotrichia*, *A vaginae*, BVAB1, UU-2 and *U parvum* as explanatory variables. Odds ratios (OR) adjusted for age and race were estimated using multivariable logistic regression. Analyses were performed using SAS version 9.1 for Windows.

## RESULTS

All bacteria analysed were common among women with PID. *L sanguinegens/amnionii*, *A vaginae*, BVAB1, UU-2, and *U parvum* were detected in 62%, 54%, 28%, 32% and 32% of women tested, respectively. There was a high degree of agreement between cervical and endometrial PCR (*Leptotrichia*: Phi coefficient 0.75, p<0.001; BVAB1: Phi coefficient 0.33, p<0.05; *U ureaplasma* (biovar 2): Phi coefficient 0.49, p<0.005; and *U parvum* Phi coefficient 0.31, p<0.05). All endometrial *Leptotrichia*, BVAB1, *U ureaplasma* (biovar 2) and *U parvum*-positive cases were also positive at the cervix. Seventy-seven per cent of *Leptotrichia*, 14% of BVAB1, 31% of *U ureaplasma* (biovar 2) and 13% of *U parvum* cervical positive cases were also positive in the endometrium. Although the relationship between cervical and endometrial PCR was not statistically significant, 80% of endometrial *A vaginae*-positive cases were also positive at the cervix and 15% of cervical positive cases were positive in the endometrium.

Demographic characteristics and variables comparing medical, sexual and behavioural history stratified by bacteria detected are presented in table 1. Women who tested positive for *L sanguinegens/amnionii* were significantly more likely to be black (OR 4.9, 95% CI 1.4 to 16.9) and to have douched within the last month (OR 3.1, 95% CI 1.0 to 10.2). Similarly, women who tested positive for *A vaginae* were more likely to be uninsured (OR 6.1, 95% CI 1.2 to 30.1), report a history of *Trichomonas vaginalis* (OR 9.3, 95% CI 1.1 to 80.9) and to have douched in the last month (OR 3.1, 95% CI 1.0 to 9.9). None of the characteristics analysed were statistically associated with UU-2, *U parvum*, or BVAB1. Characteristic profiles were similar to those among women with positive *M hominis* and *G vaginalis* cultures, bacteria traditionally associated with BV, although cell sizes were small and not all comparisons were statistically significant (table 2). In addition, women testing positive for *M hominis* were 18 times as likely to report a history of PID (OR 18.0, 95% CI 1.1 to 299.0).

**Table 1:** Characteristics of women with and without *L sanguinegens/amnionii*, *U urealyticum* biovar 2, *U parvum*, *A vaginae* and BVAB1 identified in the endometrium and/or cervix

Characteristic	Lepto+	Lepto−	OR (95% CI)	UU-2+	UU-2−	OR (95% CI)	UP+	UP−	OR (95% CI)	*A vaginae*+	*A vaginae*−	OR (95% CI)	BVAB1+	BVAB1−	OR (95% CI)
	n = 31	n = 19		n = 16	n = 34		n = 15	n = 32		n = 27	n = 23		n = 14	n = 36	
	No (%)	No (%)		No (%)	No (%)		No (%)	No (%)		No (%)	No (%)		No (%)	No (%)	
Demographic															
Age, years															
<25	17 (54.8)	10 (52.6)	1.1 (0.4 to 3.4)	10 (62.5)	17 (50.0)	1.7 (0.5 to 5.6)	8 (53.3)	17 (53.1)	1.0 (0.3 to 3.4)	14 (51.9)	13 (56.5)	0.8 (0.3 to 2.5)	8 (57.4)	19 (52.8)	1.2 (0.3 to 4.1)
⩾25	14 (45.2)	9 (47.4)	Ref group	6 (37.5)	17 (50.0)	Ref group	7 (46.7)	15 (46.9)	Ref group	13 (48.2)	10 (43.5)	Ref group	6 (42.9)	17 (47.2)	Ref group
Race/ethnicity															
American	23 (74.2)	7 (36.8)	4.9 (1.4 to 16.9)	12 (75.0)	18 (52.9)	2.7 (0.7 to 10.0)	9 (60.0)	19 (59.4)	1.0 (0.3 to 3.6)	19 (70.4)	11 (47.8)	2.6 (0.8 to 8.3)	9 (64.3)	21 (58.3)	1.3 (0.4 to 4.6)
White/Hispanic/other	8 (25.8)	12 (63.2)	Ref group	4 (25.0)	6 (47.1)	Ref group	6 (40.0)	13 (40.6)	Ref group	8 (29.6)	12 (52.2)	Ref group	5 (35.7)	15 (41.7)	Ref group
Education															
<High school	12 (38.7)	5 (26.3)	1.8 (0.5 to 6.2)	7 (43.8)	10 (29.4)	1.9 (0.5 to 6.4)	4 (26.7)	11 (34.4)	0.7 (0.2 to 2.7)	9 (33.3)	8 (34.8)	0.9 (0.3 to 3.0)	6 (42.9)	11 (30.6)	1.7 (0.5 to 6.1)
⩾High school	19 (61.3)	14 (73.7)	Ref group	9 (56.3)	24 (70.6)	Ref group	11 (73.3)	21 (65.6)	Ref group	18 (66.7)	15 (65.2)	Ref group	8 (57.1)	25 (69.4)	Ref group
Insured															
No	17 (77.3)	6 (50.0)	3.4 (0.8 to 15.4)	7 (63.6)	16 (69.6)	0.8 (0.2 to 3.5)	5 (50.0)	18 (81.8)	0.2 (0.0 to 1.2)	16 (84.2)	7 (46.7)	6.1 (1.2 to 30.1)	9 (75.0)	14 (63.6)	1.7 (0.4 to 8.2)
Yes	5 (22.7)	6 (50.0)	Ref group	4 (36.4)	7 (30.4)	Ref group	5 (50.0)	4 (18.2)	Ref group	3 (15.8)	8 (53.3)	Ref group	3 (25.0)	8 (36.4)	Ref group
Medical history															
History of STI	19 (61.3)	11 (57.9)	1.2 (0.4 to 3.7)	10 (62.5)	20 (58.8)	1.2 (0.3 to 4.0)	8 (53.3)	20 (62.5)	0.7 (0.2 to 2.4)	17 (63.0)	13 (56.5)	1.3 (0.4 to 4.1)	9 (64.3)	21 (58.3)	1.3 (0.4 to 4.6)
History of GC	10 (32.3)	2 (10.5)	4.1 (0.8 to 21.0)	5 (31.3)	7 (20.6)	1.8 (0.5 to 6.7)	3 (20.0)	9 (28.1)	0.6 (0.1 to 2.8)	8 (29.6)	4 (17.4)	2.0 (0.5 to 7.8)	5 (35.7)	7 (19.4)	2.3 (0.6 to 9.1)
History of CT	9 (30.0)	10 (52.6)	0.4 (0.1 to 1.3)	7 (43.8)	12 (36.4)	1.4 (0.4 to 4.6)	5 (33.3)	13 (41.9)	0.7 (0.2 to 2.5)	9 (33.3)	10 (45.5)	0.6 (0.2 to 1.9)	5 (35.7)	14 (40.0)	0.8 (0.2 to 3.0)
History of *Trichomonas*	8 (25.8)	1 (5.3)	6.3 (0.7 to 54.8)	1 (6.3)	8 (23.5)	0.2 (0.02 to 1.9)	4 (26.7)	4 (12.5)	2.5 (0.5 to 12.0)	8 (29.6)	1 (4.4)	9.3 (1.1 to 80.9)	3 (21.4)	6 (16.7)	1.4 (0.3 to 6.4)
History of BV	6 (20.0)	2 (10.5)	2.1 (0.4 to 11.8)	2 (12.5)	6 (18.2)	0.6 (0.1 to 3.6)	0 (0.0)	8 (25.8)	–	4 (14.8)	4 (18.2)	0.8 (0.2 to 3.6)	3 (21.4)	5 (14.3)	1.6 (0.3 to 8.0)
History of PID	5 (16.7)	4 (21.1)	0.8 (0.2 to 3.2)	4 (25.0)	5 (15.2)	1.9 (0.4 to 8.2)	2 (13.3)	5 (16.1)	0.8 (0.1 to 4.7)	5 (19.2)	4 (17.4)	1.1 (0.3 to 4.8)	2 (15.4)	7 (19.4)	0.8 (0.1 to 4.2)
Sexual history															
Sexually active	26 (83.8)	17 (89.5)	0.6 (0.1 to 3.5)	14 (87.5)	29 (85.3)	1.2 (0.2 to 7.0)	11 (73.3)	30 (93.8)	0.2 (0.0 to 1.1)	24 (88.9)	19 (82.6)	1.7 (0.3 to 8.5)	13 (92.9)	30 (83.3)	2.6 (0.3 to 23.8)
Rare/occasional condom use*	17 (65.4)	14 (82.4)	0.4 (0.1 to 1.8)	12 (85.7)	19 (65.5)	3.2 (0.6 to 17.0)	9 (81.8)	21 (70.0)	1.9 (0.3 to 10.8)	15 (62.5)	16 (84.2)	0.3 (0.1 to 1.4)	9 (69.2)	22 (73.3)	0.8 (0.2 to 3.4)
New sexual partner	4 (12.9)	1 (5.3)	2.7 (0.3 to 25.8)	0 (0.0)	5 (14.7)	–	2 (13.3)	3 (9.4)	1.5 (0.2 to 10.0)	4 (14.8)	7 (4.4)	3.8 (0.3 to 37.0)	3 (21.4)	2 (5.6)	4.6 (0.7 to 31.4)
Behavioural															
Douched within last month	20 (64.5)	7 (36.8)	3.1 (1.0 to 10.2)	7 (43.8)	20 (58.8)	0.5 (0.2 to 1.8)	11 (73.3)	14 (43.8)	3.5 (0.9 to 13.5)	18 (66.7)	9 (39.1)	3.1 (1.0 to 9.9)	9 (64.3)	18 (50.0)	1.8 (0.5 to 6.4)
Drug use	5 (16.1)	5 (26.3)	0.5 (0.1 to 2.2)	4 (25.0)	6 (17.7)	1.6 (0.4 to 6.5)	3 (20.0)	6 (18.8)	1.1 (0.2 to 5.1)	5 (18.5)	5 (21.7)	0.8 (0.2 to 3.3)	2 (14.3)	8 (22.2)	0.6 (0.1 to 3.2)
Current smoker	13 (41.9)	8 (42.1)	1.0 (0.3 to 3.2)	9 (56.3)	12 (35.3)	2.4 (0.7 to 7.9)	5 (33.3)	14 (43.8)	0.6 (0.2 to 2.3)	13 (48.2)	8 (34.8)	1.7 (0.6 to 5.5)	5 (35.7)	16 (44.4)	0.7 (0.2 to 2.5)

*Condoms used in 0–5 out of 10 sexual encounters. BV, bacterial vaginosis; BVAB1, bacterial vaginosis-associated bacteria type 1; CT, *Chlamydia trachomatis*; GC, gonococcus; Lepto, *Leptotrichia*; OR, odds ratio; PID, pelvic inflammatory disease; STI, sexually transmitted infection; UP, *Ureaplasma parvum*; UU-2, *Ureaplasma* *urealyticum* biovar 2.

**Table 2:** Characteristics of women with and without *M hominis* and *G vaginalis* cultured from the endometrium

Characteristic	*M hominis*+	*M hominis*−	OR (95% CI)	*G vaginalis*+	*G vaginalis*−	OR (95% CI)
	n = 3	n = 21		n = 9	n = 20	
	No (%)	No (%)		No (%)	No (%)	
Demographic						
Age, years						
<25	2 (66.7)	12 (57.1)		5 (55.6)	14 (70.0)	
⩾25	1 (33.3)	9 (42.9)	1.5 (0.1 to 19.2)	4 (44.4)	6 (30.0)	0.5 (0.1 to 2.7)
Race/ethnicity						
African American	2 (66.7)	9 (42.9)		7 (77.8)	8 (40.0)	
White/Hispanic/other	1 (33.3)	12 (57.1)	2.7 (0.2 to 34.2)	2 (22.2)	12 (60.0)	5.3 (0.9 to 32.0)
Education						
<High school	1 (33.3)	6 (28.6)		4 (44.4)	6 (30.0)	
⩾High school	2 (66.7)	15 (71.4)	1.3 (0.1 to 16.5)	5 (55.6)	14 (70.0)	1.9 (0.4 to 9.5)
Insured						
No	2 (100.0)	9 (64.3)		2 (50.0)	10 (71.4)	
Yes	0 (0.0)	5 (35.7)	–	2 (50.0)	4 (28.6)	0.4 (0.04 to 3.9)
Medical history						
History of STI	3 (100.0)	13 (61.9)	–	4 (44.4)	13 (65.0)	0.4 (0.1 to 2.1)
History of GC	1 (33.3)	5 (23.8)	1.6 (0.2 to 21.6)	2 (22.2)	4 (20.0)	1.1 (0.2 to 7.8)
History of CT	2 (66.7)	9 (42.9)	2.7 (0.2 to 34.2)	2 (22.2)	9 (45.0)	0.4 (0.1 to 2.1)
History of *Trichomonas*	2 (66.7)	3 (14.3)	12.0 (0.8 to 177.4)	3 (33.3)	3 (15.0)	2.8 (0.4 to 18.0)
History of BV	2 (66.7)	3 (14.3)	12.0 (0.8 to 177.4)	1 (11.1)	4 (20.0)	0.5 (0.1 to 5.2)
History of PID	2 (66.7)	2 (10.0)	18.0 (1.1 to 299.0)	0 (0.0)	3 (15.0)	–
Sexual history						
Sexually active	3 (100.0)	19 (90.5)	–	8 (88.9)	18 (90.0)	0.9 (0.1 to 11.3)
Rare/occasional condom use*	1 (33.3)	14 (73.7)	0.2 (0.01 to 2.4)	7 (87.5)	11 (61.1)	4.5 (0.5 to 44.4)
New sexual partner	1 (33.3)	3 (14.3)	3.0 (0.2 to 44.4)	2 (22.2)	0 (0.0)	–
Behavioural						
Douched within last month	2 (66.7)	9 (42.9)	2.7 (0.2 to 34.2)	6 (66.7)	7 (35.0)	3.7 (0.7 to 19.6)
Drug use	0 (0.0)	6 (28.6)	–	4 (44.4)	2 (10.0)	7.2 (1.01 to 20.0)
Current smoker	0 (0.0)	10 (47.6)	–	6 (66.7)	8 (40.0)	3.0 (0.6 to 15.6)

*Condoms used in 0–5 out of 10 sexual encounters. BV, bacterial vaginosis; CT, *Chlamydia trachomatis*; GC, gonococcus; OR, odds ratio; PID, pelvic inflammatory disease; STI, sexually transmitted infection.

Anaerobic bacteria, *M hominis* and *G vaginalis* were detected more often in women with *L sanguinegens/amnionii* and *A vaginae*, although only the association between *Leptotrichia* and anaerobic bacteria was statistically significant (table 3). *G vaginalis* was cultured from the endometrium significantly more often among women testing positive for *U parvum*, but not among women testing positive for UU-2. BVAB1 was not significantly associated with any of the cultivable BV pathogens considered in our analyses.

**Table 3:** Co-presence of cultivable BV-associated bacteria* among women with *L sanguinegens/amnionii*, *U urealyticum* (biovar 2), *U parvum*, *A vaginae* and BVAB1

Characteristic	Lepto+	Lepto−	p Value‡	UU-2+	UU-2−	p Value‡	UP+	UP−	p Value	*A vaginae*+	*A vaginae*−	p Value‡	BVAB1+	BVAB1−	p Value‡
	n = 19 (%)	n = 10 (%)		n = 9 (%)	n = 20 (%)		n = 10 (%)	n = 18 (%)		n = 17 (%)	n = 12 (%)		n = 8 (%)	n = 21 (%)	
Anaerobic bacteria†	42.1	0.0	0.02	33.3	25.0	0.30	40.0	22.2	0.21	41.2	8.3	0.06	37.5	23.8	0.27
*M hominis*	21.4	0.0	0.18	0.0	15.8	0.48	0.0	18.8	0.28	21.4	0.0	0.18	25.0	6.3	0.22
*G vaginalis*	42.1	10.0	0.08	33.3	30.0	0.33	60.0	16.7	0.02	41.2	16.7	0.13	37.5	28.6	0.30

*Anaerobic bacteria, *M hominis* and *G vaginalis* endometrial cultures available on a subset of 29 women. †Selected Gram-negative anaerobes include: *Prevotellabivia*, *P disiens*, *Prevotellaoralis/veroralis*, *P oulora/veroralis*, *P buccalis/oralis*, *P oris/buccae*, *Preveotella* species, *Bacteroides ureolyticus*, anaerobic GNR non-pigmented, black anaerobic negative rod, *Prevotella intermedia*, *P corporis*, *P denticola/loeschii*, *P denticola/melaninogenica*, *Porphyromonas asaccharolytica*, *P endodontails/asaccharolytica*, *Bactercides levii*, *Bacteroides fragilis*, *Bacteroides thetaiotaormicron*, *Bacteroides distasonis*, *Bacteroides ovatus*, *Bacteroides caccae*, *Bacteroides uniformis*, *Bacteroides vulgatus*, *Bacteroides merdae*, *Bacteroides*, *Fusobacterium nucleatum*, *Fusobacterium* spp, and *Veillonella* sp. Selected anaerobic Gram-positive cocci include: *P anaerobius*, *P asaccharolyticus*, *P magnus/micros*, *P prevottii*, *P tetradius*, *Peptococcusniger*, *Peptostreptococcus species* and not speciated anaerobic Gram-positive cocci. ‡Fisher’s exact. BV, bacterial vaginosis; BVAB1, bacterial vaginosis-associated bacteria type 1; Lepto, *Leptotrichia*; UP, *Ureaplasma parvum*; UU-2, *Ureaplasma* *urealyticum* biovar 2.

Women in whom *L sanguinegens/amnionii*, *A vaginae* and BVAB1 were detected were generally more likely to have each of Amsel’s criteria for BV, although not all associations were statistically significant (see table 4). All bacteria except for *U ureaplasma* (biovar 2) and *U parvum* were more likely to be categorised as having BV defined both by Amsel’s criteria (*L sanguinegens/amnionii* adjusted OR 2.9, 95% CI 0.5 to 15.7; *A. vaginae* adjusted OR 2.6, 95% CI 0.6 to 11.4; and BVAB1 adjusted OR 5.7, 95% CI 1.0 to 31.1) and by Nugent’s Gram stain criteria (*L sanguinegens/amnionii* adjusted OR 17.7, 95% CI 2.8 to 113.0; *A vaginae* adjusted OR 19.2, 95% CI 3.7 to 98.7; and BVAB1 adjusted OR 21.1, 95% CI 2.2 to 198.5). Results were generally similar to those for *M hominis* and *G vaginalis*, established BV-associated species, although cell sizes for comparisons of these cultured bacteria were small and not all comparisons were statistically significant.

**Table 4:** Associations between fastidious bacteria identified by PCR, cultured bacteria and bacterial vaginosis determined by Gram stain and Amsel’s criteria

Bacterial vaginosis variable	Positive %	Negative %	Crude OR (95% CI)	Adjusted OR* (95% CI)
*L sanguinegens/amnionii*				
Discharge	41.9	38.9	1.1 (0.4 to 3.7)	0.8 (0.2 to 2.9)
Vaginal ph >4.5	95.7	68.8	10.0 (1.0 to 96.4)	14.5 (1.2 to 173.0)
Whiff test	51.6	36.8	1.8 (0.6 to 5.9)	1.8 (0.5 to 6.6)
Clue cells >20%	63.3	31.6	3.7 (1.1 to 12.7)	5.7 (1.3 to 25.0)
BV: Amsel’s criteria	60.9	40.0	2.3 (0.6 to 8.8)	2.9 (0.5 to 15.7)
BV: Nugent’s criteria	75.0	25.0	9.0 (2.2 to 37.2)	17.7 (2.8 to 113.0)
UU-2				
Discharge	62.5	30.3	3.8 (1.1 to 13.5)	3.4 (0.93 to 12.41)
Vaginal ph >4.5	100.0	76.0	–	–
Whiff test	50.0	44.1	1.3 (0.4 to 4.2)	1.3 (0.4 to 4.7)
Clue cells >20%	43.8	54.6	0.7 (0.2 to 2.2)	0.7 (0.2 to 2.4)
BV: Amsel’s criteria	57.1	50.0	1.3 (0.4 to 5.1)	1.5 (0.3 to 6.8)
BV: Nugent’s criteria	64.3	53.3	1.6 (0.4 to 5.8)	1.7 (0.4 to 6.9)
UP				
Discharge	35.7	40.6	0.8 (0.2 to 3.0)	0.8 (0.2 to 2.9)
Vaginal ph >4.5	81.8	88.5	0.6 (0.1 to 4.)	0.4 (0.04 to 3.8)
Whiff test	33.3	50.0	0.5 (0.1 to 1.8)	0.5 (0.1 to 1.8)
Clue cells >20%	46.7	54.8	0.7 (0.2 to 2.5)	0.7 (0.2 to 2.5)
BV: Amsel’s criteria	40.0	57.7	0.5 (0.1 to 2.2)	0.4 (0.1 to 2.3)
BV: Nugent’s criteria	61.5	55.2	1.3 (0.3 to 4.9)	1.4 (0.4 to 5.3)
*A vaginae*				
Discharge	44.4	36.4	1.4 (0.4 to 4.4)	1.2 (0.35 to 3.93)
Vaginal ph >4.5	100.0	64.7	–	–
Whiff test	59.3	30.4	3.3 (1.0 to 10.8)	3.3 (1.0 to 11.3)
Clue cells >20%	66.7	31.8	4.3 (1.3 to 14.3)	4.5 (1.3 to 16.0)
BV: Amsel’s criteria	63.6	37.5	2.9 (0.8 to 11.1)	2.6 (0.6 to 11.4)
BV: Nugent’s criteria	83.3	25.0	15.0 (3.4 to 65.6)	19.2 (3.7 to 98.7)
BVAB1				
Discharge	50.0	37.1	1.7 (0.5 to 5.9)	1.7 (0.5 to 6.0)
Vaginal ph >4.5	100.0	76.9	–	–
Whiff test	78.6	33.3	7.3 (1.7 to 31.3)	8.7 (1.9 to 40.4)
Clue cells >20%	85.7	37.1	10.2 (2.0 to 52.7)	12.6 (2.2 to 71.4)
BV: Amsel’s criteria	76.9	40.0	5.0 (1.1 to 22.8)	5.7 (1.0 to 31.1)
BV: Nugent’s criteria	92.3	41.9	16.6 (1.9 to 144.2)	21.1 (2.2 to 198.5)
*M hominis*				
Discharge	66.7	35.0	3.7 (0.3 to 48.6)	3.6 (0.3 to 50.1)
Vaginal ph >4.5	100.0	84.2	–	–
Whiff test	100.0	47.6	–	–
Clue cells >20%	100.0	57.1	–	–
BV: Amsel’s criteria	100.0	100.0	–	–
BV: Nugent’s criteria	100.0	57.9	–	–
*G vaginalis*				
Discharge	33.3	26.3	1.4 (0.3 to 7.8)	1.2 (0.2 to 7.4)
Vaginal ph >4.5	85.7	82.4	1.3 (0.1 to 15.0)	0.6 (0.03 to 11.4)
Whiff test	55.6	35.0	2.3 (0.5 to 11.5)	2.7 (0.5 to 15.8)
Clue cells >20%	77.8	40.0	5.3 (0.9 to 32.0)	6.1 (0.9 to 43.3)
BV: Amsel’s criteria	71.4	31.2	5.5 (0.8 to 38.7)	17.7 (1.3 to 248.1)
BV: Nugent’s criteria	88.9	43.8	10.3 (1.0 to 102.8)	6.6 (0.6 to 69.6)

*Adjusted for age and race. BV, bacterial vaginosis; BVAB1, bacterial vaginosis-associated bacteria type 1; OR, odds ratio; UP, *Ureaplasma parvum*; UU-2, *Ureaplasma* *urealyticum* biovar 2.

## DISCUSSION

We conclude that *L sanguinegens/amnionii*, *A vaginae* and BVAB1 are associated with BV defined by Gram stain and Amsel’s criteria among women with histologically confirmed PID. Consistent results using varying BV definitions suggest robust findings. Our results agree with those reported by Ferris *et al*,^5^ in which *A vaginae* was present in 55% of BV patients compared with 8% of controls and Fredricks *et al*,^6^ in which *A vaginae*, *Leptotrichia* and BVAB were all associated with BV defined using Amsel’s criteria. The role of newly classified ureaplasma biovars in BV has not been widely studied, and to our knowledge, ours is the first to compare BV among women with and without *U parvum*. Our null finding is consistent with evidence demonstrating no relationship between *U parvum* and urethritis in men.^12^ Only one published study has examined the relationship between UU-2 and BV, reporting that among 49 women delivering preterm, UU-2 was more frequent among those with compared with those without clinically diagnosed BV (OR 15.0, 95% CI 1.2 to 209.0).^18^ Although BV by Amsel’s and Nugent’s criteria were both more common among women testing positive for UU-2, compared with women testing negative for this bacteria (57% vs 50% and 64% vs 53%, respectively), these differences were modest and not statistically different in our study.

To our knowledge, our study is the first to compare a range of clinical characteristics consistent with BV among women with and without these fastidious bacteria. Although *L sanguinegens/amnionii*, *A vaginae* and BVAB1 were all associated with BV defined using both Nugent’s and Amsel’s criteria, and elevated pH was a universal feature, other BV characteristics differed by pathogen, suggesting variable clinical presentations. Only UU-2 was associated with vaginal discharge, whereas a positive whiff test was significantly more common among women testing positive for BVAB1, and a 20% or greater classification of epithelial cells as clue cells was more common among women testing positive for *L sanguinegens/amnionii*, *A vaginae*, or BVAB1. As UU-2 has been associated with urethritis in men,^12^ ^13^ and it was associated with vaginal discharge and elevated pH but had the weakest relationship with the remaining BV characteristics, it is tempting to speculate that this bacterium could be associated with cervicitis. With the exception of *M hominis*, none of the bacteria studied were associated with a history of BV, suggesting the possibility of previous asymptomatic BV among women with *Leptotrichia*, *A vaginae* and BVAB1. The relationship between BVAB1 and clue cells is supported by a fluorescence in-situ hybridisation study published by Fredricks and colleagues,^6^ in which BVAB were found to attach to vaginal epithelial cells, similar to the clue cells characteristic of BV.

**Key messages**

- *L sanguinegens/amnionii*, *A vaginae*, bacterial vaginosis-associated bacteria type 1 (BVAB1), *Ureaplasma urealyticum* biovar 2 and *U parvum* were commonly identified in cervical and endometrial specimens collected from women with histologically confirmed endometritis.
- Fastidious anaerobic bacteria, including *L sanguinegens/amnionii*, *A vaginae* and BVAB1, were associated with clinical characteristics consistent with bacterial vaginosis (BV) and BV defined by Nugent’s and Amsel’s criteria.
- As none were associated with abnormal vaginal discharge, these anaerobes may cause unrecognised infection.
- Routine screening using targeted PCR may be warranted to prevent sequelae among asymptomatic women who would otherwise be undiagnosed.

Our findings suggest that *L sanguinegens/amnionii* and *A vaginae* may be associated with sexual activity, as characteristics associated with STI, including uninsured status, STI history and report of a new sexual partner, were associated with these bacteria, although not all comparisons were statistically significant. Data supporting sexual activity associations with ureaplasmas and BVAB were less compelling. Women testing positive for *Leptotrichia* were nearly five times as likely to be African-American, although no other bacteria were significantly associated with race. Douching was among the strongest risk factors in our study, with women harbouring *L sanguinegens/amnionii* and *A vaginae* being three times more likely to report douching, although confidence intervals included one demonstrating borderline associations. Similarly, women with BVAB1 were almost twice as likely to report douching, although this difference was not significant. These findings are consistent with evidence showing that douching, through alteration of the vaginal flora, is associated with BV.^19^

It is possible that although these novel bacteria are frequent among women with BV, they do not play a pathogenic role. A limitation of our pilot study is the small sample size and the culture of *G vaginalis* and *M hominis* in only a subset of the parent study, which did not allow adjustment for other pathogens. The associations between *Leptotrichia*, *A vaginae*, BVAB1 and BV by Gram stain may merely suggest that these bacteria frequently co-inhabit the lower genital tract of women with other BV pathogens. In fact, our study shows that *G vaginalis*, anaerobic bacteria and *M hominis* are more common among women with compared with women without *L sanguinegens/amnionii* and *A vaginae*. This supposition is supported by a study of 55 women attending a gynaecology outpatients clinic, in which 50% of *A vaginae* patients were found to co-harbour *G vaginalis*, an organism frequently identified among women with BV.^20^ In addition, biofilms composed of both *G vaginalis* and *A vaginae* are prominent among women with BV.^21^ Alternatively, *G vaginalis* may not be pathogenic, but rather a marker for infection with other fastidious bacteria, because *G vaginalis* is also frequent among women without BV^6^ and is not associated with other reproductive morbidity.

BV and BV-associated microorganisms are associated with PID,^1^ although the microbial aetiology of non-gonococcal, non-chlamydial PID is not completely characterised. As *L sanguinegens/amnionii*, *A vaginae*, BVAB1, UU-2 and *U parvum* were highly prevalent, our findings suggest that several newly recognised species may play a role in the aetiology of a significant number of non-gonococcal, non-chlamydial PID cases. Although cross-sectional, our findings also suggest that these pathogens may ascend from the lower to the upper genital tract, as evidenced by a high degree of agreement between cervical and endometrial PCR. The quest to determine novel PID pathogens is of great interest, as *Neisseria gonorrhoeae* and/or *Chlamydia trachomatis* are only recovered from approximately a third to a half of women with PID,^19^ ^22^ resulting in an indeterminate microbiological aetiology in up to 70% of PID cases. Our findings support the need for additional studies examining these fastidious BV-associated bacteria among women with and without PID. Furthermore, although we could not examine gonococcal and chlamydial co-infection in our study, the possibility that infection with fastidious BV-associated bacteria may further increase the risk of upper genital tract infection among women with chlamydia or gonorrhoea should be explored in subsequent studies.

Although evidence for a role between newly identified BV-associated organisms and PID is limited, the high prevalence of these pathogens in our study is consistent with a handful of case studies, suggesting associations between *A vaginae* and tubo-ovarian abscess,^23^ and *L sanguinegens/amnionii* and postpartum fever, endometritis, adnexal masses and fetal death.^24^^–^26 Furthermore, in a study of 45 women with salpingitis and 44 women seeking tubal ligations, broad-range 16S rDNA PCR was used to identify bacterial 16S sequences in the fallopian tube specimens from 24% of cases and no controls.^27^ Bacteria phylotypes closely related to *Leptotrichia* spp and *A vaginae* were among those identified in cases.

In summary, PID patients infected with *L sanguinegens/amnionii*, *A vaginae* and BVAB1 were more likely than women testing negative by PCR to have BV. Our study suggests that although these pathogens are associated with BV, these novel bacteria may cause unrecognised infection, as none were associated with abnormal vaginal discharge and only *Leptotrichia* was associated with a previous history of BV. Lack of vaginal discharge may hinder patient and clinical recognition of BV, suggesting that a number of cases may currently go undetected. As these bacteria have been found to have a high sensitivity and specificity for BV determined by Gram stain,^28^ routine screening using targeted PCR may be warranted to prevent sequelae among asymptomatic women who would otherwise be undiagnosed. Furthermore, current treatment regimens may be inadequate for a number of women with BV, as *A vaginae* has been found to be metronidazole resistant.^5^ As the effectiveness of currently recommended BV treatments for women with *L sanguinegens/amnionii* and BVAB is unknown, further studies are needed in order to optimise treatment and prevent sequelae.
